# Hypofractionated Image-Guided Radiation Therapy With Simultaneous-Integrated Boost Technique for Limited Metastases: A Multi-Institutional Analysis

**DOI:** 10.3389/fonc.2019.00469

**Published:** 2019-06-04

**Authors:** Corbin D. Jacobs, Manisha Palta, Hannah Williamson, Jeremy G. Price, Brian G. Czito, Joseph K. Salama, Michael J. Moravan

**Affiliations:** ^1^Department of Radiation Oncology, Duke University Medical Center, Durham, NC, United States; ^2^Radiation Oncology Clinical Service, Durham VA Medical Center, Durham, NC, United States; ^3^Department of Biostatistics and Bioinformatics, Duke University Medical Center, Durham, NC, United States

**Keywords:** simultaneous-integrated boost, oligometastasis, oligoprogression, radiotherapy, stereotactic, elective, occult, marginal recurrence

## Abstract

**Purpose:** To perform a multi-institutional analysis following treatment of limited osseous and/or nodal metastases in patients using a novel hypofractionated image-guided radiotherapy with simultaneous-integrated boost (HIGRT-SIB) technique.

**Methods:** Consecutive patients treated with HIGRT-SIB for ≤5 active metastases at Duke University Medical Center or Durham Veterans' Affairs Medical Center between 2013 and 2018 were analyzed to determine toxicities and recurrence patterns following treatment. Most patients received 50 Gy to the PTV_boost_ and 30 Gy to the PTV_elect_ simultaneously in 10 fractions. High-dose treatment volume recurrence (HDTVR) and low-dose treatment volume recurrence (LDTVR) were defined as recurrences within PTV_boost_ and PTV_elect_, respectively. Marginal recurrence (MR) was defined as recurrence outside PTV_elect_, but within the adjacent bone or nodal chain. Distant recurrence (DR) was defined as recurrences not meeting HDTVR, LDTVR, or MR criteria. Freedom from pain recurrence (FFPR) was calculated in patients with painful osseous metastases prior to HIGRT-SIB. Outcome rates were estimated at 12 months using the Kaplan-Meier method.

**Results:** Forty-two patients met inclusion criteria with 59 sites treated with HIGRT-SIB (53% nodal and 47% osseous). Median time from diagnosis to first metastasis was 31 months and the median age at HIGRT-SIB was 69 years. The most common primary tumors were prostate (36%), gastrointestinal (24%), and lung (24%). Median follow-up was 11 months. One acute grade ≥3 toxicity (febrile neutropenia) occurred after docetaxel administration immediately following HIGRT-SIB. Four patients developed late grade ≥3 toxicities: two ipsilateral vocal cord paralyzes and two vertebral compression fractures. The overall pain response rate was 94% and the estimated FFPR at 12 months was 72%. The estimated 12 month rate of HDTVR, LDTVR, MR, and DR was 3.6, 6.2, 7.6, and 55.8%, respectively. DR preceded MR, HDTVR, or LDTVR in each instance. The estimated 12 month probability of in-field and marginal control was 90.0%.

**Conclusion:** Targeting areas at high-risk for occult disease with a lower radiation dose, while simultaneously boosting gross disease with HIGRT in patients with limited osseous and/or nodal metastases, has a high rate of treated metastasis control, a low rate of MR, acceptable toxicity, and high rate of pain palliation. Further investigation with prospective trials is warranted.

## Introduction

Ever since Hellman and Weichselbaum proposed the existence of the oligometastatic state (1) as a corollary to the spectrum theory of cancer spread (2), there has been increasing interest in treating oligometastatic patients with high-dose precisely-targeted radiation (3). Recent randomized evidence demonstrates progression-free and overall survival improvements with the use of hypofractionated image-guided radiotherapy (HIGRT) to treat limited metastases (4–6). However, the optimal radiotherapy technique used to treat limited metastatic patients remains unknown.

Current radiotherapy techniques to treat oligometastases typically utilize stereotactic body radiotherapy principles including small margins and steep dose gradients (7, 8) to minimize potential toxicity of the high dose per treatment. Consistent with this approach is an avoidance of a clinical target volume (CTV) to treat nearby microscopic cancer spread. However, patterns of progression demonstrate that using this technique, recurrences typically occur in nearby structures beyond the treated target volume (9–13).

In an attempt to prevent marginal recurrence (MR) and avoid subjecting patients to another course of treatment, we investigated a simultaneous-integrated boost (SIB) technique when delivering HIGRT. We hypothesized that treating a larger elective volume (including areas at high-risk of harboring occult disease) with a lower dose considered to be well-tolerated by nearby organs at risk, while simultaneously boosting gross disease to a higher dose, would decrease MR with an acceptable toxicity profile.

## Materials and Methods

### Patient Selection

Consecutively treated patients with lymph node and/or osseous metastases treated with the HIGRT-SIB technique in the Department of Radiation Oncology at Duke University Medical Center or the Durham Veterans' Affairs Medical Center prior to October 1, 2018 were identified. Patients >18 years of age with pathologically confirmed solid tumor malignancy of any primary site with five or fewer active metastatic sites at the time of HIGRT-SIB were included in this analysis. The combination of computed tomography (CT) and nuclear medicine imaging (i.e., bone scan and/or positron emission tomography [PET] as indicated by National Comprehensive Cancer Network guidelines) were used to quantify the number of active metastatic sites prior to HIGRT-SIB. All prostate cancer patients were staged with a combination of CT scans and technetium-99 m bone scans, while all other patients were staged with a combination of CT scans and ^18^F-fluorodeoxyglucose-PET scans.

We extracted the following information from medical records: age at HIGRT-SIB, gender, primary tumor site, tumor histology, primary tumor treatment, systemic therapy, time to metastatic disease, number of active metastatic sites, largest diameter of metastasis (cm), biomarker level before and after HIGRT-SIB (i.e., prostate specific antigen [PSA], carcinoembryonic antigen [CEA], alpha-fetoprotein [AFP], carbohydrate antigen 19-9 [CA 19-9], and thyroglobulin), presence of painful metastasis prior to HIGRT-SIB, dose per fraction to PTV receiving boost dose (PTV_boost_), dose per fraction to PTV receiving elective dose (PTV_elect_), number of fractions, gross tumor volume (GTV, cm^3^), PTV_boost_ (cm^3^), PTV_elect_ (cm^3^), and date of death or last follow-Up.

### Treatment Technique

Patients were typically simulated supine with raised arms in a customized immobilization device, with respiratory management and intravenous contrast as indicated with 2–3 mm CT slices. The GTV was contoured on each axial slice. An elective CTV was contoured encompassing the gross disease and areas at high-risk of occult spread, including the surrounding nodal chain or contiguous bone. Typically, in the case of bony spine metastases, the entire vertebrae was included in the CTV as well as the spinal cord and canal at that level. The CTV was expanded by 5–7 mm in each direction to generate the PTV_elect_. The GTV was expanded by 0–5 mm in each direction to generate the PTV_boost_. Metastases with overlapping PTV_boost_ were considered as a single site, unless they involved different organs (e.g., obturator lymph node and pelvic bone).

The most frequently prescribed dose-fractionation was 50 Gy to the PTV_boost_ and 30 Gy to PTV_elect_ over 10 fractions. Organs at risk were contoured and assigned dose constraints compiled from published prospective and retrospective analyses (14–16). The PTV_boost_ could be selectively under-dosed to meet constraints of dose-limiting organs at risk such as the spinal cord, cauda equina, brachial plexus, and hollow viscera. Treatment was delivered on a linear accelerator with volumetric modulated arc therapy (VMAT) or intensity-modulated radiotherapy (IMRT) with cone beam computed tomography (CBCT) alignment approved by the physician prior to each fraction. Patients were seen once weekly during HIGRT-SIB for assessment of acute toxicity, 4–6 weeks after treatment, and then follow-up and imaging performed as clinically indicated.

### Outcomes

The primary outcome was the probability of in-field and marginal control. Events that contributed to this primary outcome include high-dose treatment volume recurrence (HDTVR), low-dose treatment volume recurrence (LDTVR), and MR. HDTVR was defined as clinical and/or radiographic progression or recurrence within the PTV_boost_. LDTVR was defined as clinical and/or radiographic recurrence within the PTV_elect_. MR was defined as clinical or radiographic recurrence outside the PTV_elect_ but within the same bone or nearby lymph node chain. Distant recurrence (DR) was defined as clinical or radiographic recurrence at a new site outside the PTV_elect_ that did not meet criteria for MR. Three authors (CJ, JS, MM) independently reviewed each clinical and radiographic recurrence, and a consensus categorization was reached in every case.

For metastases from prostate, thyroid, or gastrointestinal primaries with elevated biomarkers prior to treatment, biochemical recurrence (BR) was defined as biomarker elevation above the pre-HIGRT-SIB level. Overall survival (OS) was defined as the time from HIGRT-SIB start to death or last follow-up date. Disease-free survival (DFS) was defined as the time from HIGRT-SIB start to first recurrence (HDTVR, LDTVR, MR, DR, or BR), death, or last follow-up date, whichever was sooner.

Acute and late toxicities were measured using the Common Terminology Criteria for Adverse Events (CTCAE) version 5.0. Improvement in pain was defined as any decrease in severity on a 10-point scale after HIGRT-SIB. Pain recurrence was defined as equating or exceeding the metastasis pain severity from the pre-HIGRT-SIB level on a 10-point scale. Freedom from pain recurrence (FFPR) was calculated from the time of HIGRT-SIB start to pain recurrence in patients with painful osseous metastases prior to treatment.

### Statistical Analysis

Demographic, tumor, and treatment characteristics were summarized with N (%) for categorical variables and median (interquartile range) for continuous variables for all metastases or patients, where applicable. Median length of follow-up was calculated from the start of HIGRT-SIB until death or last contact date for all patients. Crude event rates for each of the previously defined clinical endpoints were calculated out of the applicable populations (i.e., varying denominators). For HDTVR, LDTVR, MR, and probability of in-field and marginal control rates were calculated out of the total number of metastatic sites. Probability of in-field and marginal recurrence was also stratified by whether the metastasis was nodal or osseous and groups were compared with a log-rank test. For DR, OS, and DFS, rates were calculated out of the total number of patients. For BR, the rate was calculated for the total number of patients with pre-HIBRT-SIB elevated biomarkers. For FFPR, the rate was calculated out of the total number of patients with painful osseous sites of disease. Estimates and 95% confidence intervals (CI) of 1- and 2 year rates and median time to event for clinical endpoints were estimated using the Kaplan-Meier (K-M) method. Additionally, K-M estimates of the primary endpoint were calculated for nodal vs. osseous metastases, and K-M estimates of DFS were calculated by number of active metastases at time of HIGRT-SIB. Differences in in-field or marginal recurrence or DFS by metastasis location and number, respectively, were compared between the groups using a log-rank test. Statistical analysis was performed using R version 3.4.3 (17), with Kaplan-Meier estimates obtained from the survival package (18).

## Results

### Baseline Characteristics

Between July 2013 and October 2018, 42 patients met the inclusion criteria and 59 sites were treated with HIGRT-SIB. Demographic, disease, and treatment characteristics are summarized in Table 1. Median time from diagnosis to first metastasis was 31 months, and the median age at HIGRT-SIB was 69 years. The majority of patients had a single (52%) or two (31%) active metastatic sites at the time of HIGRT-SIB. The most common primary tumor was prostatic adenocarcinoma (36%), followed by gastrointestinal (24%), and lung (24%).

**Table 1:** Demographic, tumor, and treatment characteristics.

**Patient-specific variable (*n* = 42)**	**N (%) or median** **(IQR)**
Age at HIGRT-SIB	69 (60–72)
Gender	
Female	8 (19)
Male	34 (81)
Primary tumor site	
Gastrointestinal^*^	10 (24)
Kidney	1 (2)
Head and neck^°^	2 (5)
Lung^†^	10 (24)
Prostate	15 (36)
Skin	3 (7)
Testicle^‡^	1 (2)
Histology	
Adenocarcinoma	27 (64)
Follicular dendritic cell sarcoma	1 (2)
Hepatocellular carcinoma	2 (5)
Melanoma	2 (5)
Merkel cell carcinoma	1 (2)
Mesothelioma^‡^	1 (2)
Papillary thyroid with follicular features	1 (2)
Renal cell carcinoma	1 (2)
Small cell carcinoma	2 (5)
Squamous cell carcinoma	4 (10)
Time from diagnosis to first metastasis (months)	31 (5–103)
Number of active metastases at time of HIGRT-SIB	
1	22 (52)
2	13 (31)
3	5 (12)
4	0 (0)
5	2 (5)
Biomarker level prior to HIGRT-SIB (*n* = 23)	
AFP (ng/mL)	29 (5–53)
CA19-9 (U/mL)	26^**^
CEA (ng/mL)	8.8 (5.9–11.0)
PSA (ng/mL)	8.9 (2.5–12.0)
Thyroglobulin (μg/L)	216^**^
**Treated metastasis-specific variable (*****n*** **=** **59)**	**N (%) or median** **(IQR)**
HIGRT-SIB target	
Lymph node metastasis	31 (53)
Painful osseous metastasis	16 (27)
Non-painful osseous metastasis	12 (20)
HIGRT-SIB anatomic location	
Abdominopelvic	27 (46)
Spine	14 (24)
Sternum or rib	8 (14)
Supraclavicular fossa, mediastinum, or axilla	10 (17)
Greatest diameter of largest metastasis (cm)	3.0 (2.1–3.7)
GTV (cm^3^)	12.4 (4.9–20.9)
PTV_boost_ (cm^3^)	30.0 (15.5–49.7)
PTV_elect_ (cm^3^)	182.7 (108.2–315.7)
Dose to PTV_elect_	30 (30–30)
Fractions	10 (10–10)
HIGRT-SIB duration, days	13 (11–14)

* *The distribution among gastrointestinal primary tumors was one anal canal, three colorectal, three esophagus, two liver, and one periampullary*.

° *One patient had papillary thyroid cancer with follicular features and another had p16-negative squamous cell carcinoma of the tonsil*.

† *One patient had medically inoperable oligometastatic extrapulmonary small cell carcinoma and comorbid contraindications to systemic therapy. After HIGRT-SIB, this patient remains disease-free for over 30 months. One patient had follicular dendritic cell sarcoma and received HIGRT-SIB to five sites per multidisciplinary consensus recommendations in lieu of systemic therapy*.

‡ *One patient had oligometastatic testicular mesothelioma*.

** *Single measurement*.

*AFP, Alpha-fetoprotein; CA 19-9, carbohydrate antigen 19-9; CEA, carcinoembryonic antigen; GTV, gross tumor volume; HIGRT-SIB, hypofractionated image-guided radiotherapy with simultaneous-integrated boost; IQR, interquartile range; PTV, planning target volume; PSA, prostate specific antigen*.

Among the 59 sites treated with HIGRT-SIB, 53% were nodal and 47% were osseous. Nearly one-half (46%) of all nodal or osseous metastases were in an abdominopelvic site, and nearly one-quarter (24%) were in the cervical, thoracic, or lumbar spine. The median GTV and PTV_boost_ were 12.4 and 30.0 cm^3^, respectively. The median net PTV enlargement to generate the PTV_elect_ was 164 cm^3^ with respect to PTV_boost_. All but four patients received a prescribed dose of 50 and 30 Gy in 10 fractions to the PTV_boost_ and PTV_elect_, respectively. Three patients were selectively underdosed to meet spinal cord or brachial plexus constraints, and one patient received 30 and 20 Gy in five fractions to the PTV_boost_ and PTV_elect_, respectively.

### Toxicity and Pain Analysis

Table 2 summarizes the acute and late toxicities per treated site or course of HIGRT-SIB. The most common acute toxicities were grade 1–2 fatigue (55%) and grade 1–2 gastrointestinal (42%). An acute pain flare occurred in four osseous sites (14%) and no nodal sites. Patients requiring a short course of steroids or non-steroidal anti-inflammatory drugs to facilitate laying comfortably on the treatment table during HIGRT were considered acute grade 1–2 neurologic toxicities (10%). The incidence of all other acute grade 1–2 toxicities was <10%, including dermatitis (8%). One acute grade ≥3 toxicity was noted in a patient who received docetaxel immediately following HIGRT-SIB and was subsequently hospitalized for febrile neutropenia. No other acute grade ≥3 toxicities were noted.

**Table 2:** Acute and late toxicities per treated site of HIGRT-SIB (*n* = 59).

**Toxicity**	**Acute grade** **1–2** ***N* (%)**	**Acute grade** **≥3** ***N* (%)**	**Late grade** **1–2** ***N* (%)**	**Late grade** **≥3** ***N* (%)**
Fatigue^*^	26 (55)	0 (0)	0 (0)	0 (0)
Gastrointestinal	25 (42)	0 (0)	6 (10)	0 (0)
Genitourinary	3 (5)	0 (0)	0 (0)	0 (0)
Hematologic^*^	2 (4)	1 (2)^†^	0 (0)	0 (0)
Musculoskeletal	0 (0)	0 (0)	2 (3)	2 (3)^‡^
Neurologic	6 (10)	0 (0)	0 (0)	2 (3)^°^
Respiratory	2 (3)	0 (0)	0 (0)	0 (0)
Skin	5 (8)	0 (0)	0 (0)	0 (0)

* *Rates were reported per course of HIGRT-SIB (n = 47)*.

† *Febrile neutropenia 5 weeks after completing HIGRT-SIB in a single patient with prostate cancer treated with HIGRT-SIB to two pelvic sites immediately followed by a cycle of docetaxel*.

‡ *Two patients developed ipsilateral vocal cord paralysis*.

° *One patient required kyphoplasty for compression fracture and one patient required long-term narcotics for vertebral compression fracture limiting activities of daily living*.

*HIGRT-SIB, hypofractionated image-guided radiotherapy with simultaneous-integrated boost*.

Late grade ≥3 toxicity following HIGRT-SIB was noted in four patients. Two of these were vertebral compression fractures; one requiring kyphoplasty and another treated with long-term narcotics for pain that limited the patient's activities of daily living. The two other grade ≥3 toxicities occurred in patients with esophageal cancer treated to the supraclavicular fossa and/or upper mediastinum who developed ipsilateral vocal cord paralysis. One of these patients had hoarseness prior to HIGRT-SIB, and underwent multiple esophageal dilations for grade 2 dysphagia. The other patient manifested hoarseness 32 months after completing HIGRT-SIB that did not improve with vocal cord injection. Of note, both of these patients had received prior thoracic chemoradiation therapy for their primary disease and one of the two patients underwent subsequent esophagectomy.

There were 12 patients with painful bony metastases in the study and 11 of them reported pain relief following treatment. The estimated 12 month FFPR was 72%. In total, there were 16 painful osseous metastatic sites treated, with 15 (94%) noted as having a decrease in severity following treatment.

### Patterns of Recurrence

**Figure 1:**
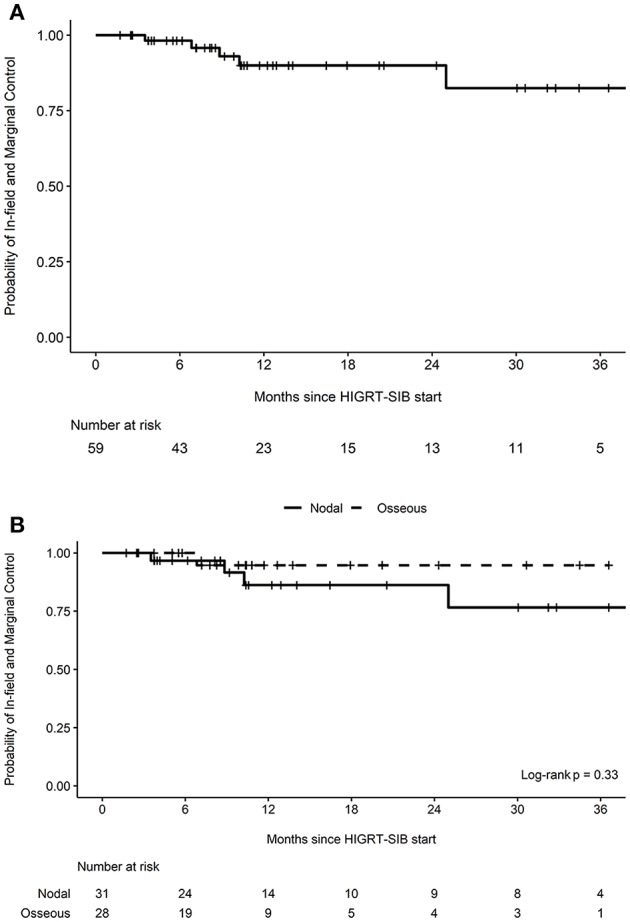
Probability of in-field and marginal control. Kaplan-Meier curves depicting the probability of in-field and marginal control **(A)** for all treated metastatic sites (*n* = 59) as well as **(B)** stratified by nodal vs. osseous metastases treated with HIGRT-SIB.

After a median follow-up of 11 months (interquartile range 6–24 months), there were five marginal or in-field recurrences (Table 3). The estimated probability of in-field and marginal control at 12 months was 90.0% (95% CI 80.9-100.0%, Figure 1A). When stratified by whether a nodal or osseous metastasis was treated with HIGRT-SIB, the estimated probability of in-field and marginal control at 12 months was 86.2% (95% CI 72.5-100.0%) for nodal metastases and 94.7% (95% CI 85.2-100.0%) for osseous metastases (*p* = 0.33, Figure 1B).

**Table 3:** Crude and estimated rates of clinical endpoints.

**Variable**	**HDTVR**	**LDTVR**	**MR**	**BR**	**DR**	**Any recurrence**	**Death**
Crude events, *n* (%)	2/59 (3%)	1/59 (2%)	2/59 (3%)	11/23 (48%)	21/42 (50%)	26/42 (62%)	8/42 (19%)
Estimated rate at 12 months (95% CI)	3.6% (0.0–10.2%)	6.2% (0.0–17.4%)	7.6% (0.0–18.1%)	43.4% (18.1–60.9%)	55.8% (31.3–71.5%)	60.1% (38.5–74.2%)	11.9% (0.0–22.5%)

*BR, Biochemical recurrence; CI, confidence interval; DR, distant recurrence; HDTVR, high dose treatment volume recurrence; LDTVR, low dose treatment volume recurrence; MR, marginal recurrence*.

**Figure 2:**
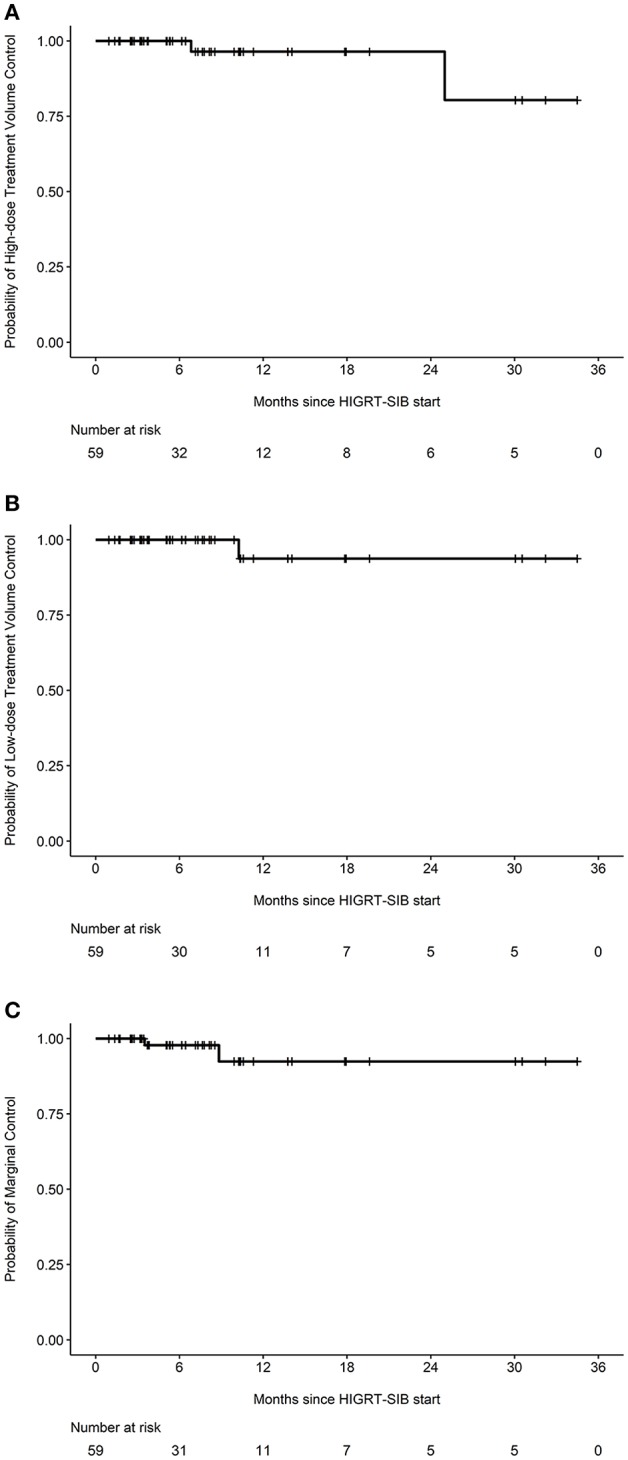
Specific probabilities of in-field and marginal control. Kaplan-Meier curves depicting the probabilities of **(A)** high-dose treatment volume control, **(B)** low-dose treatment volume control, or **(C)** marginal control among all treated metastatic sites (*n* = 59).

After review of individual isodose lines, daily CBCT, and diagnostic surveillance imaging, the crude number of events for HDTVR, LDTVR, MR, and DR were 2, 1, 2, and 21, respectively (Table 3). The estimated rates of HDTVR, LDTVR, MR, and DR at 12 months were 3.6% (95% CI 0.0-10.2%, Figure 2A), 6.2% (95% CI 0.0-17.4%, Figure 2B), 7.6% (95% CI 0.0-18.1%, Figure 2C), and 55.8% (95% CI 31.3-71.5%), respectively. The median time to DR was 11 months, and DR preceded HDTVR, LDTVR, or MR in each instance.

Further exploring MR, one occurred in a patient with lower extremity melanoma initially treated with wide local excision and inguinal nodal dissection who later received HIGRT-SIB for ipsilateral external and common iliac lymph node oligometastases. A biopsy-proven recurrence developed in the surgically dissected inguinal region, which was not included in the PTV_elect_.

**Figure 3:**
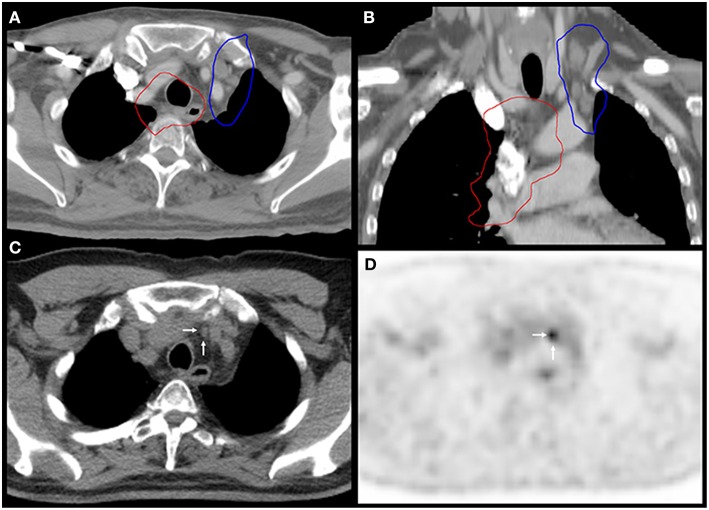
Marginal recurrence between the junctions of radiation fields. Images **(A,B)** show the 60 Gy isodose lines (red) from the first course of chemoradiation and the 30 Gy isodose lines (blue) from the PTV_elect_ of HIGRT-SIB on fused axial and coronal planning CT images. Images **(C,D)** correspond to the axial slices of the surveillance PET-CT scan that identified the marginal recurrence (white arrows) in an undertreated lymph node between the junctions of the radiation fields.

Additionally, a lung cancer patient developed both MR and LDTVR following two separate courses of HIGRT-SIB. This patient initially received 60 Gy to the primary lung tumor and mediastinal lymph nodes with concurrent carboplatin and paclitaxel. The first course of HIGRT-SIB targeted an isolated left upper mediastinal nodal recurrence while attempting to avoid overlap with the initial fields. As depicted in Figure 3, the MR occurred just outside of the PTV_elect_, right between the junctions of the radiotherapy fields. The second HIGRT-SIB course treated the right supraclavicular fossa, where a LDTVR likely occurred due to an insufficiently treated subcentimeter oligometastasis that was visible on CT, but not avid on pre-treatment positron emission tomography (PET).

Both HDTVRs occurred in patients with prostate cancer. One patient with castrate-resistance developed widespread osseous metastases on the initial surveillance scan and shortly thereafter demonstrated disease progression within the PTV_boost_ 6 months after HIGRT-SIB. The second HDTVR occurred 25 months following HIGRT-SIB in one of five treated para-aortic lymph nodes in the setting of chronic immunosuppression and a new primary bladder malignancy.

Of the 23 patients with biochemically-detectable malignancies, 11 met our definition of BR. The median time to BR was 18 months. Among those with BR, the elevated laboratory value preceded any clinical or radiographic recurrence in 73% of patients.

### Survival Analysis

Eight deaths occurred during the follow-up period (Table 3). The median OS was 36.6 months and the estimated 12 month OS was 88.1% (95% CI 77.5-100.0%). Any recurrence occurred in 26 (62%) patients during the follow-up period. The median DFS was 8.3 months and the estimated 12 month DFS was 38.8% (95% CI 25.1-60.1%). When stratified by number of active metastatic sites, the median DFS for patients with 1, 2, 3, and 5 active metastatic sites prior to HIGRT-SIB was 11.3, 7.7, 3.7, and 5.4 months, respectively.

## Discussion

Despite increasing enthusiasm for and growing evidence supporting the treatment of limited or “oligo” metastases with radiation, the optimal radiotherapy technique is unknown. Most of the evidence supporting the use of radiation for limited metastases has been accomplished using small fields directed at gross disease with a minimal margin to decrease the likelihood for toxicity. However, as progression near treated tumors occurs at a significant rate, we sought to decrease the likelihood of such by including an elective, lower-dose volume including adjacent areas at high-risk of harboring occult disease. With this technique, we found high treated tumor control rates, consistent with prior reports using HIGRT (11, 13, 19–37). Additionally, we found that the HIGRT-SIB technique altered previously reported patterns of progression, as we saw few in-field or marginal recurrences (10% combined at 12 months). Furthermore, treatment was well-tolerated with low rates of acute and late grade ≥3 toxicity, and pain responses following HIGRT-SIB were higher than historical rates observed following standard, palliative radiation doses.

The high rate of treated tumor control (96% at 12 months) seen in our patients treated with this HIGRT-SIB technique was promising. Our results are comparable with reported 12 month local control rates in other studies of HIGRT in oligometastastic patients with spinal (>80%) (20, 28, 33, 36, 38, 39), non-spine bony (>91%) (19, 22, 25, 26, 34), and lymph node metastases (>77%) (11, 13, 21, 23, 24, 29–32, 35, 37), treated to visible tumor only. Additionally, it appears that the inclusion of a low dose PTV_elect_ may have reduced nearby progression. Prior studies describing patterns of progression following HIGRT to nodal metastases report 26–55% recurrence rates in adjacent lymph nodes (11, 13, 25). For patients with spinal metastases treated with HIGRT, prior studies have described the patterns of progression occurring primarily in the epidural space and/or in adjacent bony elements that have either not been included in the treatment volumes or purposely underdosed in order to meet spinal cord constraints (9, 33, 38, 39). Of the limited studies specifically investigating MR in radiation treated spinal metastases, one reported a crude MR rate of 12.5% and a cumulative incidence at 12 months of 9.5% (10). We noted only two MRs and a single LDTVR, corresponding to a combined estimated rate of 5% at 12 months. Comparison of these rates with prior studies investigating the use of HIGRT for patients with non-spine bony metastases is difficult, as the rates and patterns of progression immediately outside of the treated field are not commonly reported in the existing, limited literature for these patients.

This HIGRT-SIB technique was well-tolerated, as both acute and late grade ≥3 toxicity rates were low (≤ 10%). Importantly, we did not observe any bowel obstruction, bowel perforation, gastrointestinal hemorrhage, myelopathy, or death due to HIGRT treatment. Comparison of our observed toxicity rate is difficult due to the heterogeneity of treatment sites included in this analysis. However, our results compare favorably with existing reports, including a recently reported prospective randomized trial of standard of care (SOC) treatment with HIGRT vs. SOC alone in patients with 1–5 metastatic sites that found 29% experienced acute grade ≥2 toxicity with three treatment-related grade 5 events (6). Another recent randomized trial of HIGRT vs. maintenance chemotherapy in oligometastatic NSCLC patients reported a 20% grade three treatment-related toxicity rate in the radiation arm (4).

We found that HIGRT-SIB resulted in a high subjective pain response (>90%) that was also durable, with 72% of patients reporting continued pain improvement at 12 months. The pain response rate seen in our study was higher than the rate of 66% reported for all patients treated on the multi-fraction palliative radiotherapy arm of RTOG 97-14 (40) as well as the rate of 62% in patients with spine metastases in that trial (41). Our results also compare favorably to reported pain response rates following stereotactic body radiotherapy (SBRT) for spinal metastases (41–98%) (38, 42–44) and non-spine bony metastases receiving SBRT (77–88%) (34, 45).

While others have used a SIB technique to treat limited metastases, our technique is novel in several ways. First, patients in this study were most commonly received 10 fractions, delivering an established oligometastatic treatment dose of 50 Gy along with an elective dose of 30 Gy, the latter of which is commonly utilized to treat metastatic disease in multiple anatomic sites. Additionally, for osseous spinal metastases we included the entire involved vertebrae, including the posterior elements and spinal canal, in the PTV_elect_. For non-spine osseous metastases a generous elective volume could be included and for patients with limited lymph node metastases, we targeted occult spread throughout the contiguous lymph node chain and not just the immediate vicinity around the involved node. We were able to treat large volume oligometastases with this technique. Finally, we were able to deliver treatments using commonly available CBCT image-guidance and without more advanced spine SBRT techniques, indicating that many centers may be able to adopt our HIGRT-SIB technique as a tool to treat oligometastatic, oligorecurrent, or oligoprogressive patients in their clinic.

Prior reports of a SIB technique for spinal metastases have utilized a CTV that included the vertebral body and selected, but not all, posterior elements in order to meet spinal cord constraints delivering one fraction of 21–24 Gy to the GTV and 18 Gy electively, or alternatively three fractions delivering 30 Gy to the GTV and 24 Gy electively (20). For lymph node metastases, HIGRT with a SIB technique utilizing 1–5 fractions with a much smaller low-dose CTV as a 5-mm expansion from gross disease with anatomic modifications has been used (46). Recently, a spinal simultaneous integrated boost (SSIB) technique for patients with spinal metastases considered to be “radiation-resistant” and unsuitable for treatment with standard spine SBRT approaches has been described (47). The study involved 12 patients with 15 treated sites extending between 3 and 5 vertebral body levels that were treated using a 10-fraction SSIB technique. Gross disease was prescribed 40 Gy whereas a CTV including the involved vertebral bodies, at-risk paraspinal space, and spinal canal was prescribed 30 Gy. The 1 year local control rate was 93%, which was similar to our analysis. While only 78% of patients in this study reported any improvement in metastasis-related pain, 94% of the treated sites in our analysis resulted in any improvement in pain, potentially reflecting a dose-response relationship. Although the HIGRT-SIB technique and SSIB techniques have many similarities, there are several distinct differences. First, we used a higher dose per fraction to treat our GTV, which may have accounted for the improved pain response. Second, our CTV only included para-spinal areas if there was evidence of extraosseous extension on diagnostic imaging. Lastly, the SSIB technique was specifically utilized in patients with spinal metastases unfit for standard HIGRT, while this study described HIGRT-SIB use for patients with non-spine bony and lymph node metastases.

There are several limitations to our retrospective analysis. First, it is subject to effects from unidentified, potentially confounding variables in this very heterogeneous population. Second, the number of patients is small and the duration of follow-up is short, and therefore may not adequately capture all late toxicities and recurrences. Finally, a major limitation is the lack of a control arm (e.g., HIGRT without SIB and an elective treatment volume) to compare the rates of marginal and treated site recurrence with the experimental HIGRT-SIB technique; however, efforts are currently underway to identify patients treated with standard HIGRT at our centers and further analyses will be forthcoming.

In conclusion, targeting areas at high-risk of occult disease by treating a larger elective volume while simultaneously boosting gross disease with HIGRT in patients with limited osseous and/or nodal metastases has acceptable rates of acute and late toxicity with low rates of marginal or in-field recurrence. HIGRT-SIB showed a high rate of overall pain response that was durable. Further investigation with a prospective trial is warranted to determine if HIGRT-SIB with a PTV_elect_ has similar rates of local control, decreases MR, improves DFS, lengthens systemic therapy-free intervals or delays switching systemic therapies, and/or results in a similar or better toxicity profile compared to standard HIGRT. Importantly, prospective trials are indicated to determine if the described HIGRT-SIB technique increases the overall palliative pain response rate and/or provides more durable pain relief in patients with osseous metastases compared to traditional palliative external beam radiotherapy.

## Data Availability

The datasets generated for this study are available on request to the corresponding author.

## Ethics Statement

The retrospective research was completed under a protocol approved by the Duke University Medical Center Institutional Review Board (Pro00101071) and a protocol approved by the Durham VA Health Care System Institutional Review Board (MIRB #1740). Research was conducted under waivers of consent, HIPAA authorization, and decedent notification at both institutions.

## Author Contributions

CJ, JS, and MM contributed conception and design of the study and organized the database. CJ and HW performed the statistical analysis. CJ wrote the first draft of the manuscript. MM, JS, HW, BC, JP, and MP wrote sections of the manuscript. All authors contributed to manuscript revision, read, and approved the submitted version.

### Conflict of Interest Statement

The authors declare that the research was conducted in the absence of any commercial or financial relationships that could be construed as a potential conflict of interest. The handling editor declared a past co-authorship with the authors JS and MM.
